# Systemic steroid administration combined with intratympanic steroid injection in the treatment of a unilateral sudden hearing loss prognosis prediction model: A retrospective observational study

**DOI:** 10.3389/fneur.2022.976393

**Published:** 2022-09-20

**Authors:** Hao Yuan, Cheng-Cheng Liu, Peng-Wei Ma, Jia-Wei Chen, Wei-Long Wang, Wei Gao, Pei-Heng Lu, Xue-Rui Ding, Yu-Qiang Lun, Lian-Jun Lu

**Affiliations:** Department of Otolaryngology Head and Neck Surgery, Tangdu Hospital, Fourth Military Medical University, Xi'an, China

**Keywords:** sudden hearing loss, risk factors, prognosis, outcome prediction, steroids

## Abstract

Idiopathic sudden sensorineural hearing loss (ISSNHL) is an emergency ear disease that is referred to as a sensorineural hearing loss of at least 30 dB in three sequential frequencies and occurs over a period of < 72 h. Because of its etiology, pathogenesis, and prognostic factors, the current treatment methods are not ideal. Previous studies have developed prognostic models to predict hearing recovery from ISSNHL, but few studies have incorporated serum biochemical indicators into previous models. The aim of this study was to explore the factors influencing the ISSNHL prognosis of combination therapy (combined intratympanic and systemic use of steroids, CT), among the patient population data, the serum biochemical indicators before the treatment, and the clinical features of ISSNHL. The new prediction model was developed through these factors. From November 2015 to April 2022, 430 patients who underwent CT at the Department of Otorhinolaryngology Head and Neck Surgery, Tangdu Hospital, Air Force Medical University for ISSNHL, were reviewed retrospectively. We found significant differences in age (*P* = 0.018), glucose (*P* = 0.035), white blood cell (WBC) (*P* = 0.021), vertigo (*P* = 0.000) and type (*P* = 0.000) with different therapeutic efficacies. Multivariate logistic regression analysis showed that age (OR = 0.715, *P* = 0.023), WBC (OR = 0.527, *P* = 0.01), platelet to lymphocyte ratio (PLR) (OR = 0.995, *P* = 0.038), vertigo (OR = 0.48, *P* = 0.004), course (time from onset to treatment) (OR = 0.681, *P* = 0.016) and type (OR = 0.409, *P* = 0.000) were independent risk factors for ISSNHL prognosis. Based on independent risk factors, a predictive model and nomogram were developed to predict hearing outcomes in ISSNHL patients. The area under the curve (AUC) value of the model developed in this study was 0.773 (95% CI = 0.730–0.812), which has a certain predictive ability. The calibration curve indicated good consistency between the actual diagnosed therapeutic effectiveness and the predicted probability. The model and nomogram can predict the hearing prognosis of ISSNHL patients treated with CT and can provide help for medical staff to make the best clinical decision. This study has been registered with the registration number ChiCTR2200061379.

## Introduction

Idiopathic sudden sensorineural hearing loss (ISSNHL) is referred to as a sensorineural hearing loss of at least 30 dB in three sequential frequencies and occurs over a period of < 72 h (1). As a common disease of otorhinolaryngology, ISSNHL is a common ear emergency, and its annual incidence is ~5–30/100,000, which has shown an increasing trend year by year (2). Thus far, the etiology and pathogenesis of ISSNHL remain unclear. It is currently thought to be associated with viral infection, environmental factors, occupational factors (such as exposure to noise, heavy metals and organic solvents), autoimmune diseases, cardiovascular diseases, accidents, metabolic diseases and other factors (3).

The treatment methods of ISSNHL include systemic steroid therapy (SST), intratympanic steroid injection (ITSI), combination therapy (combined intratympanic and systemic use of steroids, CT), hyperbaric oxygen therapy, antiviral drugs, thrombolysis, vasodilators or vasoactive substances and other drugs. Currently, there is no widely accepted standard treatment. The strength of evidence for the efficacy of therapeutic methods is limited to retrospective studies and only a few prospective studies. A randomized, three-blind, controlled trial found that SST had no significant effect on ISSNHL compared with placebo (4). A previous study found that ITSI significantly improved patients' hearing outcome compared with placebo (5). Then researchers combined the two treatments and found that patients treated with CT had a better hearing prognosis than those treated with SST (6). Through a meta-analysis, Han et al. found that the efficacy of CT in the treatment of ISSNHL was superior to that of SST alone (7). In a retrospective study, Skarzyńska et al. (8) found that the hearing recovery rate of ISSNHL treated by CT was higher than that of SST and ISI. In conclusion, CT is an effective treatment for ISSNHL.

To predict the efficacy of CT in the treatment of ISSNHL, Kawamura et al. predicted hearing outcomes at all frequencies in ISSNHL patients after CT, and the factors included age, time from onset to treatment, presence or absence of vertigo, hearing status before treatment and the hearing status of the healthy side. However, due to the limited predictive variables and relatively narrow prediction interval, the established model has practical value only in predicting the prognosis of high-frequency hearing in ISSNHL patients (9). Based on the time between symptom onset and treatment, the initial hearing level of both ears, BMI, age and previous history of hearing loss, Uhm et al. established a prognostic model through machine learning to estimate the prognosis of patients receiving CT. Nonetheless, because only demographic data and clinical features of ISSNHL were added to the model and the number of cases was relatively limited, the practical value of the model is unknown (10). Although there are some predictive models, few studies have taken serum biochemical parameters into account. Whether the established model is suitable for clinical application is still unknown.

In the present study, we hope to explore the influencing factors of sex, age and other demographic data of patients, biochemical, cellular and coagulation indicators at admission, and clinical features related to ISSNHL on the prognosis of ISSNHL after CT. Based on the results, a new model was established to predict the prognosis of ISSNHL.

## Materials and methods

### Patients

This study is a retrospective study. A total of 653 inpatients admitted to the Department of Otolaryngology, Head and Neck of Tangdu Hospital under ISSNHL from November 2015 to April 2022 were recruited.

### Inclusion criteria

- Aged from 18 to 84.
- Unilateral sudden deafness >30 dB at least three consecutive frequencies within 72 h for unknown reasons.
- Did not receive glucocorticoid treatment before admission.
- Only CT was used during hospitalization.

### Exclusion criteria

- Middle ear lesions, posterior cochlear space occupation, Meniere's disease and large vestibular aqueduct syndrome.
- Genetic factors and other pathogenic factors.
- Pregnant women and contraindications of glucocorticoid use.
- Incomplete medical records.

**Figure 1:**
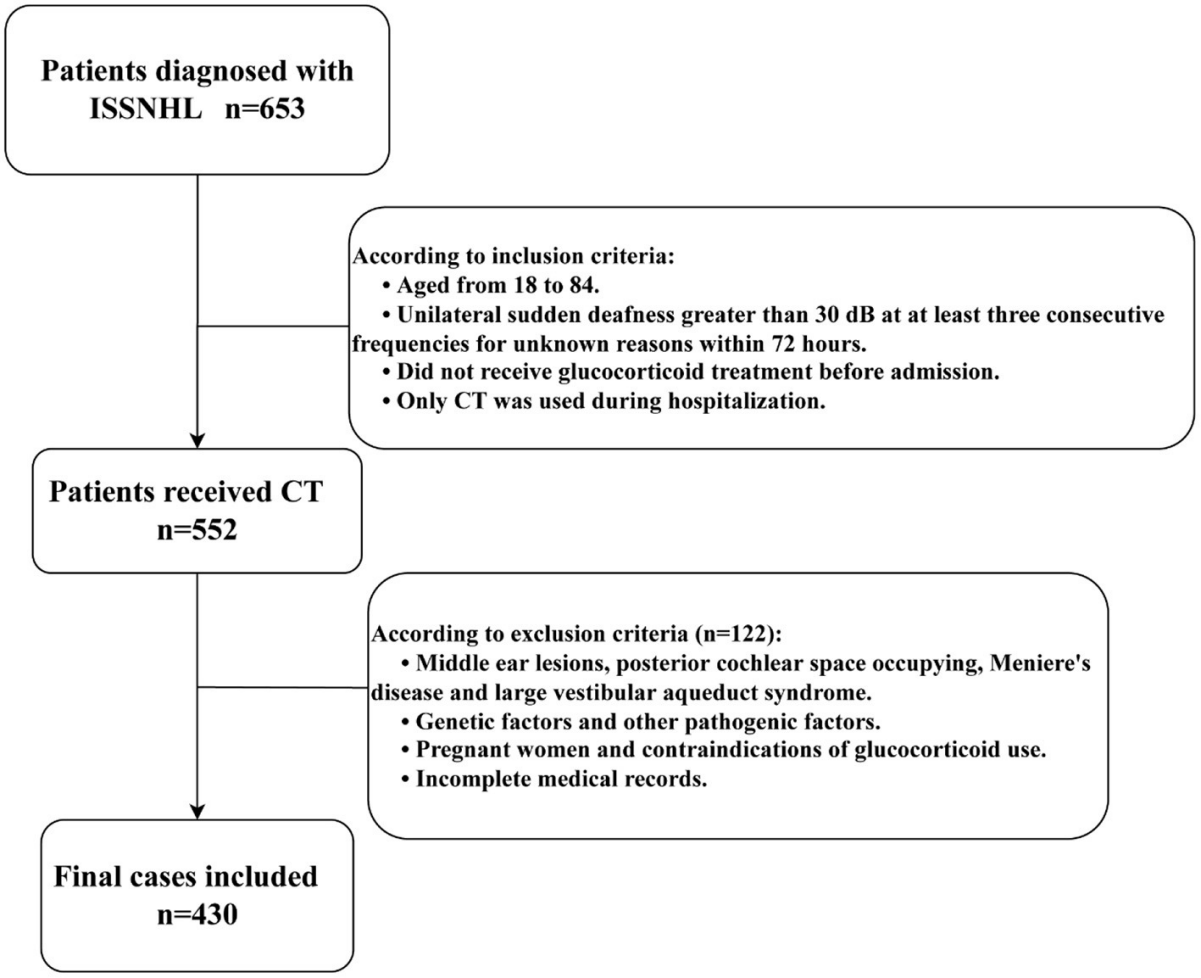
Flow chart.

Finally, 430 people were included in this study. The investigation was conducted in accordance with the criteria set out in the Declaration of Helsinki and as depicted in Figure 1, approved by the Medical Ethics Committee of Tangdu Hospital.

### Treatment protocol

All patients received systemic steroid administration combined with intratympanic steroid injection with the same protocol. SSTs were taken orally at 1 mg/kg prednisone acetate, with the maximum dose not exceeding 60 mg. They were taken orally on an empty stomach from days 1 to 5 and reduced by 10 mg daily from day 6 until drug withdrawal. The ITSI surgery was as follows: after surface anesthesia, methylprednisolone 40 mg was injected into the lower quadrant of the posterior tympanic membrane and was injected 3–4 times in total. After administration, the patient was instructed to remain in the lateral decubitus position with the affected ear facing up for at least 30 mins.

### Audiometric assessment

The pure-tone hearing thresholds from 125 to 8,000 Hz were measured at every octave before and after treatment. According to the initial hearing, ISSNHL is divided into the following four categories: ascending (the average threshold at 0.25–0.5 kHz was 20 dB higher than the average threshold at 4–8 kHz), descending (the average threshold at 4–8 kHz was 20 dB higher than the average threshold at 0.25–0.5 kHz), flat (similar threshold observed across the entire frequency range and hearing threshold not exceeding 90 dB HL) and profound (the average thresholds at 0.5, 1, 2, and 4 kHz over 90 dB HL) (11).

The mean pure tone threshold of 0.5, 1, 2 and 4 kHz after treatment was calculated. Hearing improvement was assessed using a modification of Siegel's criteria (12). Treatment ineffective was classified as meeting the following criteria: (1) average auditory threshold improvement of 10–30 dB HL; (2) mean auditory threshold improvement < 10 dB HL. A total of 310 patients failed to receive treatment. The treatment was classified as effective if the following criteria were met: (1) the mean hearing threshold returned to < 25 dB HL; (2) the mean auditory threshold improvement was >30 dB HL. The treatment was effective in 120 patients.

### Data collection

The baseline data were obtained from medical records. We collected demographic data, serum biochemical markers, and clinical features of ISSNHL. The demographic data included sex, age, body mass index (BMI), and hypertension. The serum biochemical parameters included red blood cells (RBC), white blood cells (WBC), platelets (PLT), lymphocytes, monocytes, neutrophils, neutrophil to lymphocyte ratio (NLR), platelet to lymphocyte ratio (PLR), triglycerides (TG), total cholesterol (TC), high-density lipoprotein (HDL), low-density lipoprotein (LDL), glucose, activated partial thromboplastin time (APTT), fibrinogen (Fib) and fibrin degradation product (FDP). The clinical characteristics of ISSNHL included the incentives, location, tinnitus, aural fullness, vertigo, course (time from onset to treatment), and type (shape of the audiogram).

### Statistical analysis

SPSS 26.0 statistical analysis software (IBM, Armonk, NY, USA) was used for statistical analysis, and GraphPad 8.0 (Insightful Science, USA) was used for graphing. Continuous variables are expressed as the mean (SD) and were compared using an unpaired Student's t test or Mann-Whitney test. Categorical variables were analyzed using the chi-squared test or Fisher's exact probability test. Age, sex, TG, TC, WBC, neutrophil, lymphocyte, monocyte, PLT, HDL, NLR, PLR, APTT, Fib, vertigo and tinnitus and other factors were analyzed by univariate logistic regression. Variables with *P* < 0.1 were included in multivariate logistic regression analysis. The multiple logistic regression model was built by stepwise selection using the criteria of *P* < 0.05 for selecting variables and *P* > 0.10 for backward elimination. The nomogram was formulated based on the results of multivariate logistic regression analysis using the RMS package of R, version 3.1.1 (http://www.r-project.org/). The nomogram can proportionally convert each regression coefficient in the logistic regression to a scale of 0 to 100 points. The effect of the variable with the highest beta coefficient (absolute value) is assigned 100 points. The points are then added across independent variables to derive the total points, which are converted to predicted probabilities. Making receiver operating characteristic (ROC) curves. The predictive performances of the models were measured by the area under the curves (AUCs) and calibrated with 1,000 bootstrap samples to minimize the overfitting bias. *P* < 0.05 was considered statistically significant.

## Result

### Patient baseline data

A total of 430 patients with unilateral ISSNHL were enrolled retrospectively, and their medical records were reviewed. Of these patients, 120 responded to treatment, and 310 did not. There were significant differences in age (*P* = 0.018), glucose (*P* = 0.035), WBC (*P* = 0.021), vertigo (*P* = 0.000) and type (*P* = 0.000) (Table 1). There were no significant differences in sex (*P* = 0.837), BMI (*P* = 0.361), hypertension (*P* = 0.968), incentives (*P* = 0.374), ISSNHL location (*P* = 0.904), tinnitus (*P* = 0.639), aural fullness (*P* = 0.521), TC (*P* = 0.373), TG (*P* = 0.916), HDL (*P* = 0.483), LDL (*P* = 0.533), lymphocytes (*P* = 0.779), neutrophils (*P* = 0.13), monocytes (*P* = 0.493), PLTs (*P* = 0.09), RBCs (*P* = 0.759), APTT (*P* = 0.645), Fibs (*P* = 0.917), FDPs (*P* = 0.25), NLR (*P* = 0.203), PLR (*P* = 0.137) and course (*P* = 0.05) in the patients with different therapeutic effects (Table 1).

**Table 1:** Baseline data of the patients.

**Variable**	**Effective (*n* = 120)**	**Ineffective (*n* = 310)**	**Test value**	***P* value**
**Age, y**				
18–29	25 (20.8%)	43 (13.9%)	10.031	0.018
30–44	45 (37.5%)	89 (28.7%)		
45–65	47 (39.2%)	157 (50.6%)		
>65	3 (2.5%)	21 (6.8%)		
**Sex**				
Male	61 (50.8%)	161 (51.9%)	0.042	0.837
Female	59 (49.2%)	149 (48.1%)		
**BMI, kg/m** ^**2**^				
< 18.5	4 (3.3%)	14 (4.5%)	2.039	0.361
(18.5, 24.0)	64 (53.3%)	142 (45.8%)		
(24.0, 40)	52 (43.3%)	154 (49.7%)		
**Hypertension**				
Yes	8 (6.7%)	21 (6.8%)	0.002	0.968
No	112 (93.3%)	289 (93.2%)		
**TG, mmol/L**				
< 1.7	80 (66.7%)	205 (66.1%)	0.011	0.916
≥1.7	40 (33.3%)	105 (33.9%)		
**TC, mmol/L**				
< 5.18	103 (85.8%)	255 (82.3%)	0.793	0.373
≥5.18	17 (14.2%)	55 (17.7%)		
**HDL, mmol/L**				
≤ 1.15	68 (56.7%)	164 (52.9%)	0.493	0.483
>1.15	52 (43.3%)	146 (47.1%)		
**LDL, mmol/L**				
< 3.37	110 (91.7%)	278 (89.7%)	0.388	0.533
≥3.37	10 (8.3%)	32 (10.3%)		
**Glucose, mmol/L**				
≤ 6.1	107 (89.2%)	250 (80.6%)	4.457	0.035
>6.1	13 (10.8%)	60 (19.4%)		
**WBC**, ** × 10**^**9**^**/L**				
≤ 7	73 (60.8%)	150 (48.4%)	5.368	0.021
>7	47 (39.2%)	160 (51.6%)		
**Lymphocyte**, ** × 10**^**9**^**/L**				
≤ 2	64 (53.3%)	170 (54.8%)	0.079	0.779
>2	56 (46.7%)	140 (45.2%)		
**Neutrophil**, ** × 10**^**9**^**/L**				
≤ 4	62 (51.7%)	135 (43.5%)	2.297	0.13
>4	58 (48.3%)	175 (56.5%)		
**Monocyte**, ** × 10**^**9**^**/L**				
≤ 0.35	29 (24.2%)	85 (27.4%)	0.47	0.493
>0.35	91 (75.8%)	225 (72.6%)		
**RBC**, ** × 10**^**9**^**/L**				
≤ 4.5	55 (45.8%)	137 (44.2%)	0.094	0.759
>4.5	65 (54.2%)	173 (55.8%)		
**PLT**, ** × 10**^**9**^**/L**				
≤ 200	60 (50%)	127 (41.0%)	2.872	0.09
>200	60 (50%)	183 (59.0%)		
**APTT, sec**				
≤ 28.8	88 (73.3%)	234 (75.5%)	0.213	0.645
>28.8	32 (26.7%)	76 (24.5%)		
**Fib, g/L**				
≤ 2.6	90 (75.0%)	234 (75.5%)	0.011	0.917
>2.6	30 (25.0%)	76 (24.5%)		
**FDP, ug/ml**				
≤ 1	38 (31.7%)	81 (26.1%)	1.325	0.25
>1	82 (68.3%)	229 (73.9%)		
**Incentives**				
Yes	7 (5.8%)	12 (3.9%)	0.789	0.374
No	113 (94.2%)	298 (96.1%)		
**Location**				
Left	60 (50.0%)	153 (49.4%)	0.014	0.904
Right	60 (50.0%)	157 (50.6%)		
**Tinnitus**				
Yes	113 (94.2%)	288 (92.9%)	0.22	0.639
No	7 (8.1%)	22 (7.1%)		
**Aural fullness**				
Yes	84 (70.0%)	207 (66.8%)	0.412	0.521
No	36 (30.0%)	103 (33.2%)		
**Vertigo**				
Yes	38 (31.75)	181 (58.4%)	24.716	0.000
No	82 (68.3%)	129 (41.6%)		
**Course, d**				
< 7	71 (59.2%)	144 (46.5%)	5.985	0.05
(7, 14)	26 (21.7%)	97 (31.3%)		
≥14	23 (19.2%)	69 (22.3%)		
**Type**				
Ascending	23 (19.2%)	4 (1.3%)	59.926	0.000
Descending	2 (1.7%)	5 (1.6%)		
Flat	51 (42.5%)	97 (31.3%)		
Profound	44 (36.7%)	204 (65.8%)		
**NLR, median (IQR)**	2.028 (1.29)	2.193 (1.72)	17,128	0.203
**PLR, median (IQR)**	103.22 (57.53)	108.68 (63.47)	16,883	0.137

### Univariate and multivariate analysis

The results of univariate logistic regression are presented in Table 2. Age (*P* = 0.002), glucose (*P* = 0.037), WBC (*P* = 0.021), NRL (*P* = 0.034), PLT (*P* = 0.091), PLR (*P* = 0.064), vertigo (*P* = 0.000), course (*P* = 0.065) and type (*P* = 0.000) were analyzed in the multivariate logistic regression analysis to identify independent risk factors affecting prognosis.

**Table 2:** Univariate logistic regression analysis of the treatment effectiveness.

**Variable**	**OR (95% CI)**	***P* value**
Sex	0.957 (0.628–1.459)	0.837
Age	0.671 (0.518–0.868)	0.002
BMI grade	0.856 (0.594–1.233)	0.404
Hypertension	0.983 (0.423–2.284)	0.983
TG	0.976 (0.625–1.526)	0.916
TC	0.765 (0.424–1.381)	0.374
HDL	0.859 (0.562–1.31)	0.483
LDL	0.79 (0.375–1.661)	0.534
Glucose	0.506 (0.267–0.961)	0.037
WBC	0.604 (0.267–0.961)	0.021
Neutrophils	0.722 (0.473–1.101)	0.13
Lymphocyte	1.062 (0.696–1.621)	0.779
NLR	0.876 (0.774–0.99)	0.034
Monocyte	1.185 (0.729–1.929)	0.493
RBC	0.936 (0.613–1.429)	0.759
PLT	0.694 (0.454–1.06)	0.091
PLR	0.996 (0.992–1.00)	0.064
APTT	1.12 (0.693–1.81)	0.645
Fib	1.026 (0.63–1.671)	0.917
FDP	0.763 (0.482–1.21)	0.25
Incentive	1.538 (0.591–4.006)	0.378
Location	0.975 (0.639–1.485)	0.904
Tinnitus	1.233 (0.513–2.967)	0.64
Aural fullness	1.161 (0.736–1.833)	0.521
Vertigo	0.33 (0.211–0.516)	0.000
Course	0.772 (0.587–1.017)	0.065
Type	0.383 (0.288–0.509)	0.000

Multivariate logistic regression analysis showed that age (OR = 0.715, *P* = 0.023), WBC (OR = 0.527, *P* = 0.010), PLR (OR = 0.995, *P* = 0.038), vertigo (OR = 0.480, *P* = 0.004), course (OR = 0.681, *P* = 0.016) and type (OR = 0.409, *P* = 0.000) were independent risk factors for the prognosis of ISSNHL (Table 3).

**Table 3:** Multivariate logistic regression analysis of treatment effectiveness.

**Variable**	**β**	**OR**	**95% CI**		***P* value**
			**Lower limit**	**Upper limit**	
Age	−0.335	0.715	0.536	0.954	0.023
WBC	−0.641	0.527	0.324	0.858	0.010
PLR	−0.005	0.995	0.991	1.000	0.038
Vertigo	−0.734	0.480	0.292	0.788	0.004
Course	−0.384	0.681	0.499	0.930	0.016
Type	−0.894	0.409	0.300	0.557	0.000

### Development and validation of a therapeutic effectiveness nomogram

**Figure 2:**
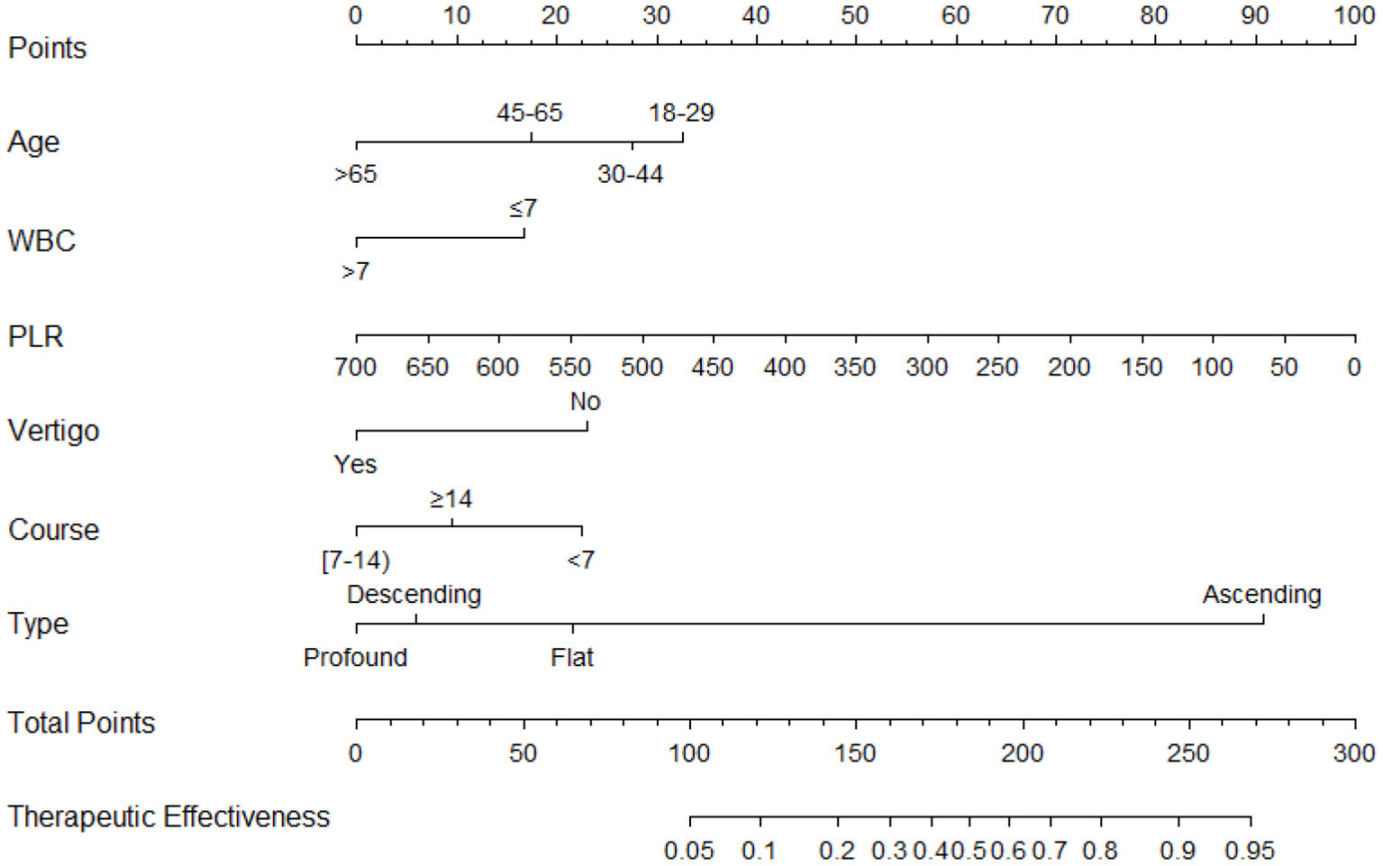
Nomogram for predicting the therapeutic effectiveness of intratympanic injection combined with systemic therapy in patients with unilateral idiopathic sudden sensorineural hearing loss. Age, year; WBC, white blood cell, × 10^9^/L; PLR, platelet to lymphocyte ratio; Course, day.

**Figure 3:**
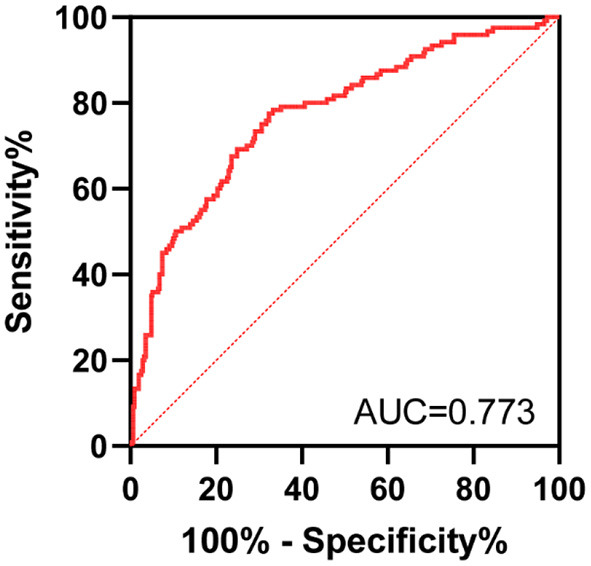
The ROC analysis for the predictive model.

**Figure 4:**
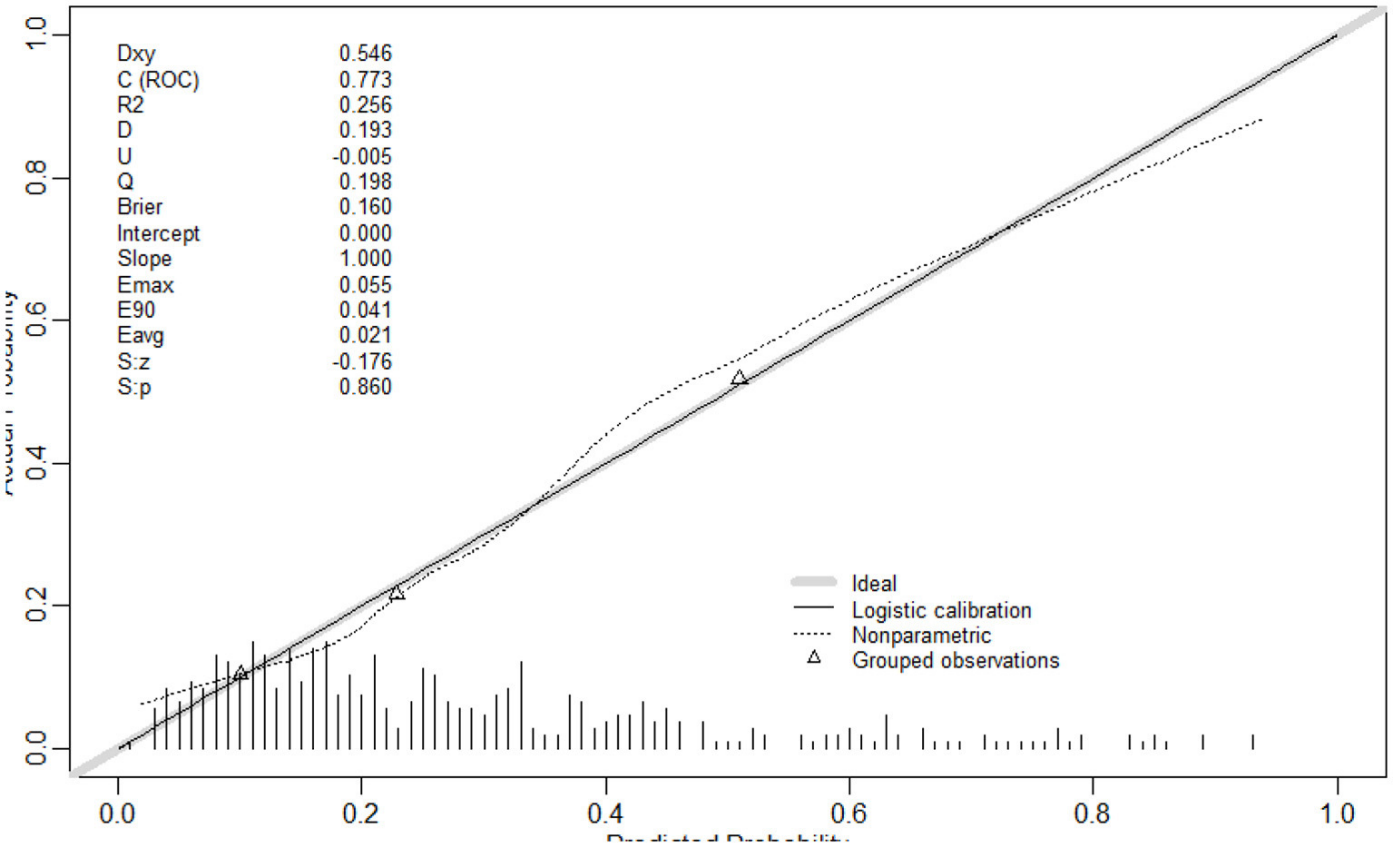
The calibration curve indicated good consistency between the actual diagnosed therapeutic effectiveness and the predicted probability.

The results of multivariate logistic regression analysis were used to establish a nomogram predicting the prognosis of CT for ISSNHL (Figure 2). The corresponding scores on the upper dots of each variable graph in the nomogram were summed to obtain the total score, and then a straight line was drawn at the bottom of the chart to estimate the therapeutic effectiveness of CT. This is helpful to evaluate the efficacy of CT as a treatment for ISSNHL. The results show that the model has high prediction accuracy and discrimination. The AUC value was 0.773 (95% CI = 0.730–0.812) (Figure 3). In addition, in the internal validation of Bootstrap, the corrected AUC was 0.778. In addition, the calibration curve showed good agreement between the predictive risk and the actual probability (Figure 4).

## Discussion

In this study, we analyzed 430 patients with unilateral ISSNHL, and investigated whether demographic characteristics, biochemical parameters and clinical features of ISSNHL could predict ISSHL hearing prognosis before CT. Furthermore, we developed and validated a predictive clinical nomogram of CT in the treatment of ISSNHL.

Demographic characteristics, clinical characteristics, and serum biochemical parameters were analyzed. In a retrospective study, Ceylan et al. (13) found that sex was an influential factor for hearing prognosis in ISSNHL patients, and female patients had a worse hearing prognosis than men. Weiss et al. (14) thought that hearing recovery was higher in men than in women. However, this study found that there was no significant difference in hearing prognosis among patients of different sexes. We believe that further clinical studies with a higher level of evidence are needed to validate this result. Suzuki et al. (15) suggested that hearing prognosis was negatively correlated with age. The results of this study showed that the hearing prognosis was different in ISSNHL patients of different ages, and the hearing prognosis worsened with the increasing age.

This study found that there is no significant difference in the effect of course on hearing prognosis. Attanasio et al. (16) believed that with the extension of the course in ISSNHL patients, the success rate of hearing prognosis decreased gradually. We believed that the reason for this result was that the chi-square test only considered the course of disease itself which did not take other factors into account. Other confounding factors of subjects with different treatment outcomes were not controlled. When using multivariate logistic regression analysis, we took multiple factors into consideration and found that the course of disease was still the influencing factor of hearing prognosis. This study found that the hearing prognosis was different with the ISSNHL type. Xie et al. found in a retrospective study that in the hearing recovery group, the proportion of patients with the ascending type and flat type was higher than that of the group without hearing recovery, and the proportion of patients with profound type was lower than that of the group without hearing recovery. The hearing prognosis of patients with different ISSNHL types was different (17). We believe that this phenomenon is related to the different pathogeneses of ISSNHL classification (18); for example, the ascending type may be hydrolabyrinth, the descending type may be hair cell damage, the flat type is more likely to be vascular stria dysfunction or inner ear vasospasm, and the profound type is most likely to be vascular embolism or thrombosis of the inner ear.

On admission, all of the ISSNHL patients had serum biochemical tests, such as WBC, RBC, PLR, NLR and glucose, performed before treatment. Wu et al. (19) believed that NLR was a predictor of hearing prognosis in ISSNHL. Contrary to the results of this study, we found that the effect of NLR on the prognosis of ISSNHL hearing was not statistically significant. Ni et al. (20) suggested that PLR can be used to predict hearing outcomes in ISSNHL patients. The results of this study are the same. Glucose and WBC count affect hearing prognosis in ISSNHL patients. Some serum biochemical indices can affect the hearing prognosis of ISSNHL patients. Therefore, we believe that it is important to include serum biochemical indices in model construction.

ISSNHL patients often have tinnitus and vertigo, and some researchers believe that the complications of ISSNHL will affect the prognosis of hearing. Previous studies have reported that tinnitus is related to hearing prognosis in ISSNHL patients (21, 22). This result is contrary to our findings. We found that whether ISSNHL patients had tinnitus had no significant effect on hearing prognosis. Bogaz et al. (23) found in a prospective cohort study that vertigo led to poorer hearing prognosis in ISSNHL patients. However, Bulgurcu et al. (24) believed that vertigo had no effect on hearing prognosis. In this study, the presence or absence of vertigo had a statistically significant effect on hearing prognosis in ISSNHL patients. Vertigo may lead to poor hearing prognosis.

Few researchers have reported a predictive model of hearing prognosis in ISSNHL. Bing et al. conducted a single-center retrospective study using multiple deep learning techniques, such as deep belief network, traditional logistic regression, support vector machine and multilayer perceptron to establish relevant ISSNHL hearing prediction models. Furthermore, they compared the prediction efficiency of different prediction models. The deep belief network had the highest prediction efficiency when it contained 149 variables. However, when the prediction model only contained three variables, such as initial hearing, course and ISSNHL type, logistic regression had the highest predictive efficiency (25). In daily clinical work, the simultaneous inclusion of 149 variables has limited clinical practicality and has high requirements for clinicians. When only 3 variables are included, the model is more conducive to the clinical application and prediction of early hearing prognosis.

In this study, 27 variables were selected. Using the forward method of multivariate binary logistic regression, we found that age, WBC, PLR, vertigo, course, and ISSNHL type were independent risk factors for the prognosis of ISSNHL hearing. The AUC value of the model developed in this study was 0.773 (95% CI = 0.730–0.812), which has a certain predictive ability. The correction curve also shows that the predicted results are in good agreement with the actual results.

There are some limitations in this study. First, all data are retrospective, and there are many confounding factors that are difficult to control. Second, this study only carried out prediction model construction and internal verification, but did not carry out external verification. Therefore, it is necessary to conduct external verification of this model in future studies to explore the feasibility of this model. Finally, prospective studies are still needed to further confirm the reliability of the nomogram.

## Conclusion

Age, WBC, PLR, vertigo, course and ISSNHL type were used as six predictive variables after taking full account of the demographic characteristics, serum biochemical indices and ISSNHL clinical characteristics. A multivariate logistic regression model and nomogram for predicting ISSNHL hearing prognosis were established. The model can predict the hearing prognosis of ISSNHL patients treated with CT and provide help for medical staff to make the best clinical decision.

## Data availability statement

The raw data supporting the conclusions of this article will be made available by the authors, without undue reservation.

## Ethics statement

The studies involving human participants were reviewed and approved by the Bioethics Committee of Tangdu Hospital of Air Force Military Medical University. Written informed consent for participation was not required for this study in accordance with the national legislation and the institutional requirements.

## Author contributions

L-JL designed the research. HY, C-CL, P-WM, J-WC, W-LW, WG, P-HL, X-RD, and Y-QL prepared the material and collected data and analyzed the data. HY prepared the figures. HY, C-CL, P-WM, and L-JL wrote the first draft of the manuscript. All authors contributed to the study conception and design. All authors commented on previous versions of the manuscript. All authors read and approved the final manuscript.

## Funding

This work was supported by funding from the Military Logistics Research Project of China (18CXZ015).

## Conflict of interest

The authors declare that the research was conducted in the absence of any commercial or financial relationships that could be construed as a potential conflict of interest.

## Publisher's note

All claims expressed in this article are solely those of the authors and do not necessarily represent those of their affiliated organizations, or those of the publisher, the editors and the reviewers. Any product that may be evaluated in this article, or claim that may be made by its manufacturer, is not guaranteed or endorsed by the publisher.
